# Genome Sequence of a Potent Biosurfactant-Producing Bacterium, *Franconibacter* sp. Strain IITDAS19

**DOI:** 10.1128/mra.00419-22

**Published:** 2022-07-18

**Authors:** Jyoti Sharma, Yogesh Kalakoti, Preeti Srivastava, Durai Sundar

**Affiliations:** a Department of Biochemical Engineering and Biotechnology, Indian Institute of Technology Delhi, New Delhi, India; b Yardi School of Artificial Intelligence, Indian Institute of Technology Delhi, New Delhi, India; Montana State University

## Abstract

Here, we report the whole-genome sequence of *Franconibacter* sp. strain IITDAS19, a potent biosurfactant-producing bacterium that was isolated from oil-contaminated soil. The sequence provided information on the genes and enzymes responsible for the biosynthesis of the biosurfactant.

## ANNOUNCEMENT

*Franconibacter* sp. strain IITDAS19 was isolated from an oil-contaminated soil sample from Assam oil fields, India (1). The bacterium is Gram negative and belongs to the family *Enterobacteriaceae*. Recently, we showed that the bacterium produces a potent biosurfactant rhamnolipid, resulting in enhanced oil recovery in simulated sand column experiments (1). However, the genes for rhamnolipid production were yet to be identified, along with the catabolic and anabolic activities of this bacterium.

The bacterium was grown in Luria broth at 30°C overnight at 180 rpm. The genomic DNA was extracted using the Quick-DNA Miniprep Plus kit (Zymo Research, Irvine, CA, USA) (2). The genomic DNA was analyzed on a 0.8% agarose gel. The paired-end library was prepared using the TruSeq Nano DNA library preparation kit, and whole-genome sequencing was performed using the Illumina NextSeq 500 platform with 2 × 150-bp chemistry. Isolated genomic DNA was fragmented with a Covaris M220 sonicator (to help in generating double-stranded DNA [dsDNA] fragments with 3′ or 5′ overhangs) to obtain a fragment distribution mean of 350 bp. Using an end-repair mixture, overhangs were converted into blunt ends, followed by adapter ligation. The ligated products were selected based on size using AMPure XP beads. Selected products were amplified by PCR using the following index primers: primer 1, AGATCGGAAGAGCACACGTCTGAACTCCAGTCA; primer 2, AGATCGGAAGAGCGTCGTGTAGGGAAAGAGTGT.

FastQC v0.11.9 (3) and Trimmomatic v0.38 (4) were used to remove adapters and check for the quality of the sequencing data (Phred scores of >30). Genome assembly was a two-step process, i.e., (i) *de novo* assembly (SPAdes v3.11.1 [5]) and (ii) reference-based scaffolding (GFinisher v2.3 [6]). Franconibacter daqui was found to be the closest species by 16S RNA-based taxonomy classification performed using the Type Strain Genome Server (TYGS) (7) and NCBI BLASTn v2.12.0 (8). A Franconibacter daqui genome (GenBank accession number ASM1464427) was used for reference-based scaffolding and preparation of the final assembly. QUAST v5.0.2 (9) and Mauve v5.0.2 (10) were used to confirm the quality of the assembly. The NCBI Prokaryotic Genome Annotation Pipeline (PGAP) v5.3 (11–13) and Prokka v3.5 (14) were used for annotation. Furthermore, genome taxonomy was analyzed using average nucleotide identity (ANI) analysis v3.8.3, pairwise tetra correlation (Tetra) v3.8.3, and Tetra Correlation Search (TCS) v3.8.3 from JSpeciesWS v3.8.3 (15).

Sequencing data yielded 3,487,110 paired reads. The final genome assembly had 19 scaffolds, a genome length of 4,675,280 bp, mean coverage of 199×, and a G+C content of 56.52%, with an *N*_50_ value of 637,943 bp. The PGAP annotation resulted in 4,435 genes, including 4,348 coding DNA sequences (CDSs), 5 rRNAs, 14 noncoding RNAs, and 70 tRNAs. The 16S rRNA NCBI BLASTn search resulted in 100% query coverage and 99% genome identity with Franconibacter daqui (GenBank accession number ASM1464427). The ANI based on BLAST (ANIb) and Tetra analyses resulted in values of 99.03% and 0.99987, respectively. TCS resulted in a value of 0.99987 for Franconibacter daqui. Default parameters were used for all software unless otherwise noted.

**FIG 1:**
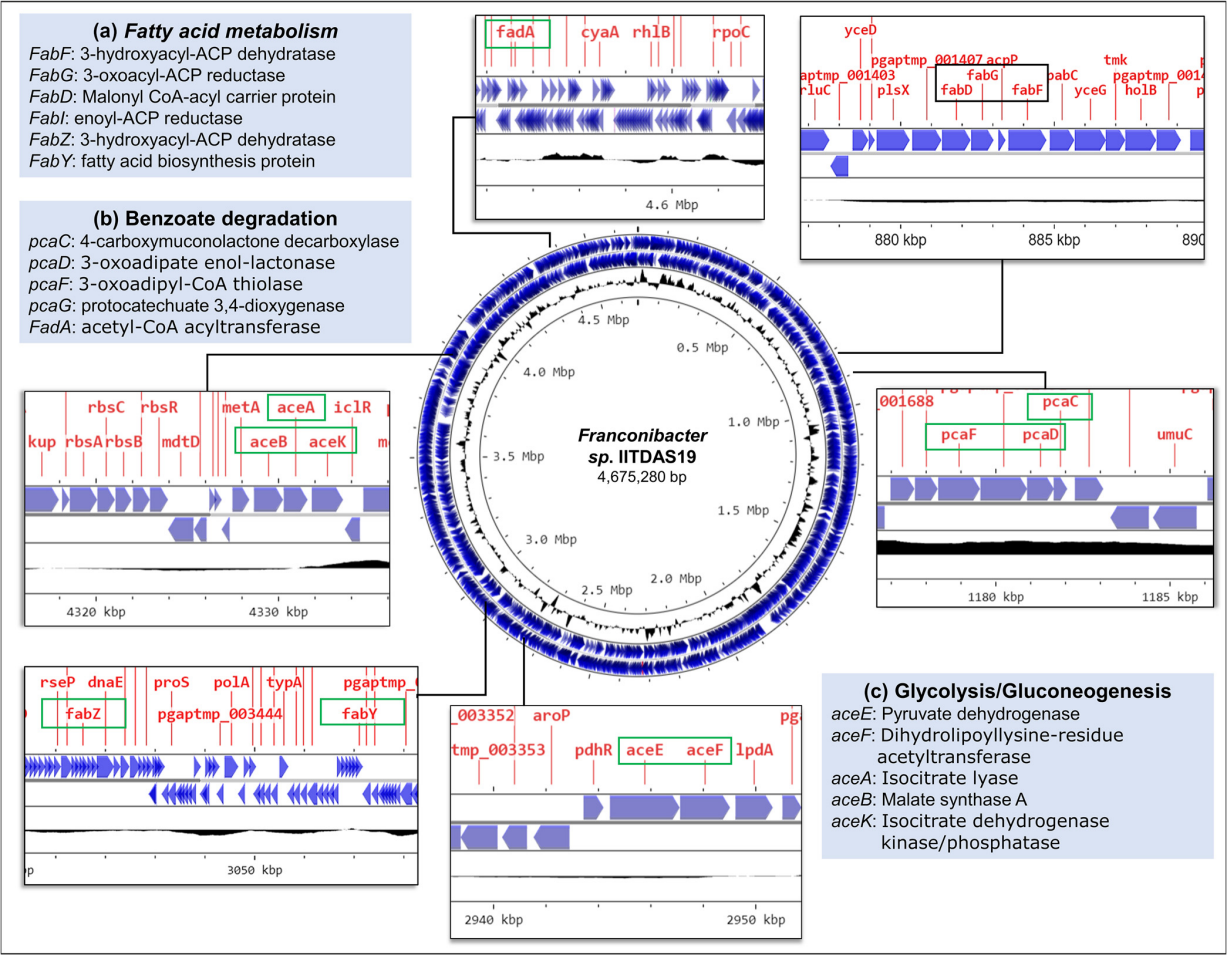
Map of the assembled *Franconibacter* sp. strain IITDAS19 genome, showing clustering of genes for major metabolic pathways such as fatty acid synthesis (a), benzoate degradation (b), and glycolysis/gluconeogenesis (c). The genes identified for these pathways are shown in green boxes.

Gene annotation analysis revealed several genes for acyltransferases and glucosyltransferases, which are essential for rhamnolipid biosynthesis. Interestingly, several genes for terpenoid and carotenoid biosynthesis were also identified in this bacterium. The genes for biodegradation of xenobiotics such as benzoate were also identified (Fig. 1).

### Data availability.

The whole-genome sequence of *Franconibacter* sp. strain IITDAS19 has been deposited in DDBJ/ENA/GenBank under the accession number JALJCP000000000, with BioProject accession number PRJNA821685, BioSample accession number SAMN27121214, and SRA accession number SRR18577966.
